# The relationship between oxidative balance scores and chronic diarrhea and constipation: a population-based study

**DOI:** 10.1186/s12889-024-18683-8

**Published:** 2024-05-21

**Authors:** Jiayan Hu, Hede Zou, Xiyun Qiao, Yuxi Wang, Mi Lv, Kunli Zhang, Fengyun Wang

**Affiliations:** 1 Xiyuan Hospital of China Academy of Chinese Medical Sciences, Beijing, 100091 China; 2 Institute of Digestive Diseases, Xiyuan Hospital of China Academy of Chinese Medical Sciences, Beijing, China; 3 Beijing University of Chinese Medicine, Beijing, China

**Keywords:** National health and nutrition examination survey, Oxidative balance scores, Constipation, Diarrhea, Mediation effect

## Abstract

**Background:**

Oxidative stress is closely related to gut health. Exposures to oxidative stress in one’s diet and lifestyle can be evaluated by the oxidative balance score (OBS). However, the relationship between OBS and intestinal habits is unknown. This study aimed to investigate the relationships between OBS and intestinal habits (chronic diarrhea and chronic constipation) and the underlying mechanisms involved.

**Methods:**

Using data from the National Health and Nutrition Examination Survey (NHANES) database from 2005 to 2010, we included a total of 8065 participants. Twenty dietary and lifestyle factors were selected for the OBS calculates. Chronic constipation and chronic diarrhea were defined using the Bristol stool form scale (BSFS) types 1 and 2 and the BSFS 6 and 7, respectively. Multivariate logistic regression, subgroup analysis, and restricted cubic splines (RCS) analysis were used to evaluate the relationship between OBS and defecation habits. Finally, we used mediation analysis to explore the indirect effects of oxidative stress and inflammatory markers on these associations.

**Results:**

After adjusting for all the covariates, multivariate logistic regression analysis revealed that OBS was negatively correlated with diarrhea (OR = 0.57; 95%CI = 0.39–0.83; *P* = 0.008)and positively correlated with constipation (OR = 1.75; 95%CI = 1.19–2.25; *P* = 0.008). The RCS showed a nonlinear relationship between OBS and diarrhea (*P* for nonlinearity = 0.02) and a linear relationship between OBS and constipation (*P* for nonlinearity = 0.19). Mediation analysis showed that the C-reactive protein (CRP) concentration and white blood cell (WBC) count mediated the correlation between OBS and diarrhea by 6.28% and 6.53%, respectively (*P* < 0.05).

**Conclusions:**

OBS is closely related to changes in patients' defecation habits. Oxidative stress and inflammation may play a role in the relationship between the two. This result emphasizes the importance of the public adjusting their lifestyle and dietary habits according to their own situation. However, further prospective studies are needed to analyze the relationship between oxidative stress and changes in defecation habits.

**Supplementary Information:**

The online version contains supplementary material available at 10.1186/s12889-024-18683-8.

## Background

Oxidative stress in organisms is caused by an imbalance between the production of reactive oxygen species (ROS) and the ability to neutralize them [1]. ROS, also known as reactive oxygen species intermediates, are byproducts of normal cellular metabolism. Usually, low-dose and medium-dose ROS have positive impacts on multiple physiological processes, such as combating invading pathogens, promoting wound healing, and promoting tissue repair. However, when the body is subjected to internal and external stimuli, excessive ROS are produced. Once a certain level is reached, peroxidation reactions of molecules such as lipids, proteins, nucleic acids, etc. are triggered. At the same time, the activity of antioxidants such as glutathione (GSH) and superoxide dismutase (SOD) decreases, leading to cellular structural damage, functional disorders, and ultimately triggering related diseases [2, 3]. As part of normal physiology, many cell types within the mucosa of the gastrointestinal tract produce ROS [4]. Enzymes that catalyze the chemical reactions that generate ROS, including NADPH oxidase, myeloperoxidase, and nitric oxide synthase, are highly expressed in the gastrointestinal tract [5]. In liver cells, ROS are produced mainly through cytochrome P450 enzymes in mitochondria and the endoplasmic reticulum [6]. However, the intestinal mucosa is also a target for a variety of oxidants that can cause pathological conditions that cause a range of digestive disorders such as gastroesophageal reflux disease, nonalcoholic fatty liver disease, gastric ulcers, and colorectal cancer [7–10]. Oxidative stress has now been found to activate inflammatory responses and improve intestinal immune responses by stimulating transcription factors, such as NF-κB, leading to intestinal injury and impaired intestinal barrier function [11]. Antioxidants can to some extent reduce the impact of oxidative stress on gastrointestinal health. For example, Shidfar F [12]. studied the dietary antioxidant index, an effective indicator reflecting total dietary antioxidant properties, and its relationship with the risk of *Helicobacter pylori* infection. They found that individuals with *H. pylori* had a lower intake of vitamin E, vitamin A, manganese, and selenium (known dietary antioxidants) were compared to controls. However, the dietary antioxidant index of the control group was greater. It is believed that appropriate intake of nutrient antibiotics may play a role in decreasing the likelihood of *H. pylori* infection. Unlike the study of individual elements, the author used the dietary antioxidant index, an indicator reflecting the overall quality of diet, to explore the relationship between antioxidant activity and disease occurrence, which is undoubtedly advanced. However, the level of oxidative stress in the body is influenced by multiple factors such as diet and lifestyle habits. We believe that exposure to a single factor or solely dietary factors may not fully reflect the body's role in maintaining overall oxidative balance, and a comprehensive evaluation of multiple factor combinations may be more meaningful. Theoxidative balance score (OBS) has a significant advantage in combining various oxidants and antioxidants in diet and lifestyle, and may be a more accurate overall indicator of oxidative stress [13]. A higher OBS reflects the advantage of antioxidants over pro-oxidant exposure, with a higher OBS indicating more antioxidant content in the body and less oxidative stress.

Chronic diarrhea and chronic constipation, common intestinal diseases, affect 17% and 20%, respectively, of the global population [14, 15], and significantly reduce the quality of life of those with these diseases. Previous studies have showed that a high intake of dietary pro-oxidants such as fats can directly stimulate the digestive tract and indirectly lead to intestinal inflammation and increased intestinal permeability through pathways such as those inducing intestinal dysbiosis [16]. ROS are important causes of intestinal diseases and are closely related to intestinal inflammation, barrier disruption, and microbial disorders [4]. Research has shown that the levels of malondialdehyde (MDA) and ROS in the liver, intestines, and blood of weaned piglets significantly increase, leading to dysbiosis of the gut microbiota and postweaning diarrhea [17]. The mechanism of action of various bacteria or viruses that can cause diarrhea, such as SARS-CoV-2 [18] and Clostridium difficile [19], is to induce excessive production of ROS, leading to oxidative stress in the body. Similarly, study has confirmed that, compared with control children, with a prolonged course of disease, the levels of vitamin C, vitamin E, the activity of SOD and catalase in the children with chronic constipation gradually decreased, while the level of lipoperoxide gradually increased. And similar results were also found in constipated mice [20, 21].

Gao Q's study revealed that, compared with that in healthy controls, the expression of NADPH oxidase (a key enzyme that produces ROS) was significantly greater in patients with chronic constipation, and it was suggested that NADPH oxidase may cause constipation by leading to dysbiosis of the gut microbiota [22]. OBS is composed of pro-oxidants (total fat, iron, alcohol intake, BMI, and cotinine) and antioxidants (dietary fiber, β-carotene, vitamin B2, niacin, vitamin B6, total folate, vitamin B12, vitamin C, vitamin E, calcium, magnesium, zinc, copper, selenium, and physical activity), and most of these elements have been proven to affect intestinal health, leading to changes in bowel habits [23–25].

There are no studies on the relationships between OBS and chronic diarrhea or chronic constipation, let alone on the pathways that may be involved in the effects of OBS on intestinal function. In addition to oxidative stress, inflammation can also affect intestinal function and play a mediating role in a large number of intestinal diseases, especially diarrhea [26]. A study by Lee et al. showed that elevated OBS levels were associated with decreased levels of inflammatory markers, including C-reactive protein (CRP) and white blood cells (WBC) [27]. Therefore, based on the above studies, we hypothesized that a low OBS concentration might reflect a high oxidative stress state in the body and further affect intestinal function by promoting the inflammatory response to trigger constipation or diarrhea.

Therefore, this study aimed to analyze the relationship between OBS and bowel habits (constipation and diarrhea) in a representative sample of the U.S. population using the National Health and Nutrition Examination Survey (NHANES). We also explored the role of oxidative stress and inflammatory markers in mediating this relationship.

## Materials and methods

### Study population

The data used in this study came from the NHANES, which is run by the Centers for Disease Control and Prevention (CDC). The NHANES is a national survey of children and adults in the U.S. that is conducted every 2 years, but information on gut health has been included for only 3 of the 2-year survey cycles (2005—2006, 2007–2008, and 2009–2010). There were a total of 31,034 participants during the three cycles; however, subjects < 20 years of age (*n* = 13902) without OBS component data (*n* = 5565) or gut health data (*n* = 2490) were excluded. In addition, 1012 participants with a self-reported history of colon cancer, pregnant women, or lack of data on covariates (mentioned later) were excluded. Overall, 8065 participants were included. The flow chart is shown in Supplementary Fig. 1.

The NHANES was approved by the Ethics Review Committee of the National Center for Health Statistics, and all participants provided written informed consent.

### Oxidative balance scores

We used the method described in the literature to calculate the OBS for each participant [28]. The OBS is calculated by summing the scores of 20 components (including 16 nutrients and 4 lifestyle components), which have been shown to be related to oxidative stress. These components can be categorized into pro-oxidants (total fat, iron, alcohol intake, BMI and cotinine) and antioxidants (dietary fiber β- carotene, riboflavin, niacin, vitamin B6, total folic acid, vitamin B12, vitamin C, vitamin E, calcium, magnesium, zinc, copper, selenium, and physical activity). In the NHANES, dietary information was obtained from participants through two 24-h dietary recall interviews (24HRs). The initial 24HR was obtained at the mobile examination center, followed by a second interview conducted via telephone 3–10 days later. These interviews aimed to capture details regarding the types and quantities of consumed food and beverages, as well as the estimated energy, nutrients, and other nutritional components. Furthermore, data on participants' use of dietary supplements were collected concurrently during both surveys. For the purpose of this study, both dietary intake and dietary supplement consumption were taken into account. Data on alcohol consumption were obtained from the question “In the past 12 months, on those days that you drank alcoholic beverages, on average, how many drinks did you have?”. Serum cotinine levels can be used to assess smoking and exposure to environmental tobacco smoke. Physical activity was represented by metabolic equivalent (MET) scores × weekly frequency of each physical activity × duration of each physical activity [29]. Physical activity includes work-related activities (intense work-related activities and moderate intensity work-related activities) and amateur physical activities (walking or cycling, intense amateur physical activities and moderate intensity amateur physical activities).

The calculation method of OBS is as follows. All components were divided into three groups by weighted tertiles. For pro-oxidants, the scores of the first, second, and third tertiles were 2, 1, and 0, respectively, while for antioxidants, the tertiles were 0, 1, and 2, respectively. OBS is obtained by adding the scores of 20 components. Because there are differences in physiology, diet and disease risk between men and women, in order to more accurately assess the state of oxidative balance, we analyzed men and women separately when calculating OBS. Table 1 lists details of the OBS component allocation scheme.

**Table 1:** Oxidative balance score assignment scheme (*n* = 8065)

**OBS components**	**Male**				**Female**		
	**Property**	**0**	**1**	**2**	**0**	**1**	**2**
Dietary OBS components							
Dietary fiber (g/d) ^a^	A	< 12.55	12.5–19.5	> 19.65	< 10.05	10.05–16.30	> 16.30
Carotene (RE/d)	A	< 98.62	98.62–305.85	> 305.85	< 98.06	98.06–383.92	> 383.92
Riboflavin (mg/d)^a^	A	< 1.79	1.79–2.69	> 2.69	< 1.34	1.34–2.02	> 2.02
Niacin (mg/d)^a^	A	< 20.64	20.64–29.75	> 29.75	< 14.51	14.51–21.85	> 21.85
Vitamin B6 (mg/d)^a^	A	< 1.59	1.59–2.40	> 2.40	< 1.13	1.13–1.77	> 1.77
Total folate (mcg/d)^a^	A	< 315.52	315.52–491.5	> 491.5	< 250.50	250.50–388.5	> 388.5
Vitamin B12 (mcg/d)^a^	A	< 3.35	3.35–6.20	> 6.20	< 2.22	2.22–4.21	> 4.21
Vitamin C (mg/d)^a^	A	< 42.40	42.40–113.15	> 113.15	< 38.00	38.00–98.40	> 98.40
Vitamin E (ATE) (mg/d)	A	< 5.82	5.82–9.41	> 9.41	< 4.53	4.53–7.52	> 7.52
Calcium (mg/d)^a^	A	< 645.50	645.50–1072.50	> 1072.50	< 499.00	499–848.50	> 848.50
Magnesium (mg/d)^a^	A	< 256.50	256.50–361.02	> 361.02	< 186.50	186.50–283.00	> 283.00
Zinc (mg/d)^a^	A	< 9.74	9.74–15.10	> 15.10	< 6.73	6.73–10.74	> 10.74
Copper (mg/d)^a^	A	< 1.12	1.12–1.57	> 1.57	< 0.85	0.85–1.28	> 1.28
Selenium (mcg/d)^a^	A	< 94.90	94.90–141.75	> 141.75	< 67.75	67.75–99.5	> 99.5
Total fat (g/d)^a^	P	> 107.44	69.8–107.44	< 69.80	> 75.81	50.94–75.81	< 50.94
Iron (mg/d)^a^	P	> 19.17	12.87–19.17	< 12.87	> 14.32	9.65–14.32	< 9.65
**Lifestyle OBS components**							
Physical activity (MET-minute/week)	A	< 415.68	415.68–1134.00	> 1134.00	< 264.13	264.13–843.27	> 843.27
Alcohol (drinks/d)	P	> 3 drinks/d	2–3 drinks/d	< = 2 drinks/d	< = 2 drinks/d	1–2 drinks/d	< = 1 drinks/day
Body mass index (kg/m2)	P	> 29.17	25.54–29.17	< 25.54	> 28.64	23.74–28.64	< 23.74
Cotinine (ng/mL)	P	> 1.13	0.04–1.13	< 0.04	> 0.17	0.04–0.17	< 0.04

*OBS* oxidative balance score, *A* antioxidant, *P* prooxidant, *RE* retinol equivalent, *ATE* alpha-tocopherol equivalent, *MET* metabolic equivalent

^a^Total intake = dietary plus supplement intakes; inclusion of supplemental intake based on the availability of supplemental intake information

### Bowel health questionnaire

According to the subjects' answers to the intestinal health questionnaire, it was determined whether they had chronic diarrhea or chronic constipation. The researchers showed participants a card with a color picture and a description of the BSFS (Type 1-type 7) as a reference. Consistent with the findings of previous studies [28, 30], subjects who identified their usual or most common stool type as BSFS type 1 (separate hard lumps, such as nuts) or BSFS type 2 (similar to sausage but massive) were classified as having chronic constipation. Patients whose usual or most common stool type was BSFS type 6 (fluffy, rough edges, paste stool) or BSFS type 7 (watery, without solid stool) were classified as having chronic diarrhea. The remaining subjects were classified as those with a normal intestinal habit.

### Intermediate targets

We selected CRP, WBC, GGT, creatinine, and 25 (OH)D as mediators to evaluate their mediating roles in the relationships between OBS and chronic diarrhea and chronic constipation. A detailed description of the laboratory method used can be found on the NHANES website. ( https://www.cdc.gov/nchs/nhanes/index.htm).

### Covariates

We adjusted for risk factors associated with chronic diarrhea and chronic constipation [31, 32]. The covariates included in this study included were age, sex, race (Mexican American, non-Hispanic white, non-Hispanic black, other Hispanic, other races), marital status (non-single, single), education (under high school, high school or equivalent, college graduate or higher), poverty-to-income ratio (PIR < 1.5, 1.5–3.5, > 3.5), milk intake (g/day), liquid intake (g/day), carbohydrate intake (g/day), sugar intake (g/day), protein intake (g/day), caffeine intake (mg/day), depression(PHQ-9 ≥ 10), and the number of combined diseases (0, 1, 2, ≥ 3) (the combined diseases included in this study included stroke, coronary heart disease, chronic kidney disease, diabetes, hypertension and hyperlipidemia). The combined disease criteria are described in the Supplemental Methods.

### Statistical analysis

All analyses weighted the samples to account for the complex sampling design of the NHANES. We used the bowel health questionnaire as a basis to calculate the weights for 6 years. Continuous variables are presented as weighted means with standard errors (means ± SDs), and categorical variables are expressed as cases (n) and weighted percentages (%). The OBS data were modeled as continuous variables and quartiles, with quartile 1 serving as the reference group. A multivariate logistic regression model was used to estimate odds ratios (ORs) and 95% confidence intervals (CIs) between OBS and chronic diarrhea and chronic constipation. We established three models: the original model was unadjusted, and model 1 was adjusted for age, sex, race, education, marital status, and PIR. Model 2 was adjusted for age, sex, race, education, marital status, PIR, milk intake (g/day), liquid intake (g/day), carbohydrate intake (g/day), sugar intake (g/day), protein intake (g/day), caffeine intake (mg/day), depression, and the number of combined diseases. The dose–response associations between OBS and the risk of chronic diarrhea and constipation were assessed using a restricted cubic spline(RCS) with a spline smoothing function. Moreover, we performed subgroup analysis to further verify the robustness of the results. To investigate the potential role of oxidative stress and inflammation in the relationship between OBS and bowel habits, we performed a simple mediation analysis to evaluate the mediating effect of the selected mediating variables on the association between OBS and bowel habits. All significance tests were two-sided, and *p* < 0.05 was used as the significance level.

## Results

### Basic characteristics of the study population

A total of 8065 participants were included in this study, including 560 with diarrhea, 529 with constipation, and 6976 healthy people. The average age of the cohort was 45.90 ± 0.38, male accounted for 50.92%, and the average OBS score was 26.09 ± 0.20. Table 2 summarize the characteristics of the participants. Univariate analysis of the diarrhea group revealed that, compared to healthy individuals, patients with diarrhea were more likely to be female, older, and have a low income, a low level of education, depression, high caffeine intake, or three or more chronic diseases. In the constipation group, compared with healthy individuals, those with constipation were more likely to be female; have a low income, a low education level, or depression; have low protein and carbohydrate intake; and have low water intake and low milk intake.

**Table 2:** Weighted characteristics of the study cohort

**Characteristics**	**Total (*n* = 8065)**	**Control (*n* = 6976)**	**Diarrhea (*n* = 560)**	***P*-value**	**Constipation (*n* = 529)**	***P*-value**
**AGE**	45.90 ± 0.38	45.82 ± 0.42	48.95 ± 0.82	< 0.001	44.18 ± 0.76	0.08
**PIR**	3.25 ± 0.04	3.28 ± 0.04	3.00 ± 0.09	0.001	2.96 ± 0.09	< 0.001
**protein (g/day)**	85.69 ± 0.58	86.60 ± 0.65	83.80 ± 2.19	0.25	74.46 ± 1.79	< 0.0001
**carbohydrate (g/day)**	262.43 ± 1.74	264.08 ± 1.78	251.60 ± 6.56	0.06	249.14 ± 4.38	0.002
**total sugars (g/day)**	118.16 ± 1.21	118.43 ± 1.19	112.87 ± 3.47	0.11	119.48 ± 3.07	0.7
**caffeine (mg/day)**	186.34 ± 4.20	186.16 ± 3.98	205.32 ± 9.87	0.03	170.36 ± 13.46	0.22
**total water (g/day)**	2054.63 ± 42.17	2085.48 ± 43.58	2054.19 ± 116.44	0.78	1609.92 ± 97.01	< 0.0001
**milk (g/day)**	596.47 ± 8.13	602.03 ± 8.18	573.12 ± 25.13	0.26	539.00 ± 23.11	0.004
**OBS. dietary**	21.81 ± 0.16	21.90 ± 0.16	21.01 ± 0.45	0.05	21.23 ± 0.46	0.1
**OBS. lifestyle**	4.28 ± 0.05	4.30 ± 0.05	3.89 ± 0.09	< 0.001	4.40 ± 0.12	0.39
**OBS**	26.09 ± 0.20	26.20 ± 0.19	24.91 ± 0.50	0.01	25.63 ± 0.56	0.24
**Gender**				< 0.001		< 0.0001
male	4227(50.92)	3815(53.07)	244(42.65)		168(28.06)	
female	3838(49.08)	3161(46.93)	316(57.35)		361(71.94)	
**Race**				0.12		0.01
Mexican American	1305( 6.66)	1100(6.45)	117(9.01)		88(7.27)	
Non-Hispanic Black	1421( 8.92)	1202(8.56)	98(9.51)		121(13.51)	
Non-Hispanic White	4415(75.81)	3888(76.46)	275(72.51)		252(69.63)	
Other Hispanic	607( 3.76)	508(3.66)	49(4.38)		50(4.68)	
Other Race	317( 4.85)	278(4.87)	21(4.60)		18(4.91)	
**Marital Status**				0.78		0.05
non-single	5127(67.53)	4474(67.89)	350(67.26)		303(62.68)	
single	2938(32.47)	2502(32.11)	210(32.74)		226(37.32)	
**Education**				< 0.0001		0.01
< high school	671( 4.05)	525(3.65)	96(9.21)		50(4.68)	
high school	2989(32.90)	2545(32.25)	215(36.64)		229(38.72)	
> high school	4405(63.05)	3906(64.10)	249(54.15)		250(56.60)	
**Depression**				< 0.0001		0.01
No	7497(94.34)	6550(95.01)	477(87.16)		470(91.69)	
Yes	568( 5.66)	426( 4.99)	83(12.84)		59(8.31)	
**Stroke**				0.02		0.52
No	7850(97.99)	6799(98.14)	537(96.17)		514(97.64)	
Yes	215( 2.01)	177(1.86)	23(3.83)		15(2.36)	
**Coronary Heart Disease**				0.35		0.12
No	7792(97.34)	6733(97.23)	542(97.88)		517(98.50)	
Yes	273( 2.66)	243(2.77)	18(2.12)		12(1.50)	
**CKD**				0.001		0.94
No	6937(89.29)	6037(89.61)	441(84.51)		459(89.49)	
Yes	1128(10.71)	939(10.39)	119(15.49)		70(10.51)	
**Diabetes**				< 0.001		0.14
No	6925(89.70)	6017(89.93)	445(83.74)		463(92.13)	
Yes	1140(10.30)	959(10.07)	115(16.26)		66( 7.87)	
**Hypertension**				0.1		0.16
No	4986(66.26)	4327(66.25)	302(61.60)		357(71.02)	
Yes	3079(33.74)	2649(33.75)	258(38.40)		172(28.98)	
**Hyperlipidemia**				0.02		0.82
No	2241(28.64)	1975(28.98)	112(23.85)		154(28.41)	
Yes	5824(71.36)	5001(71.02)	448(76.15)		375(71.59)	
**Disease State**				< 0.0001		0.46
No	1453(20.25)	1288(20.61)	62(15.16)		103(20.13)	
1	3119(41.77)	2724(41.79)	182(37.48)		213(45.68)	
2	1998(24.31)	1729(24.47)	151(25.23)		118(21.11)	
3 and more	1495(13.66)	1235(13.13)	165(22.13)		95(13.09)	

Mean ± SEs for continuous variables, *P*-values were calculated by weighted linear regression model

Percentage (%) for categorical variables, *P*-values were calculated by weighted chi-square test

*OBS* oxidative balance score, *PIR* poverty income ratio, *CKD* chronic kidney disease

### Association between OBS and chronic diarrhea and constipation

**Fig. 1:**
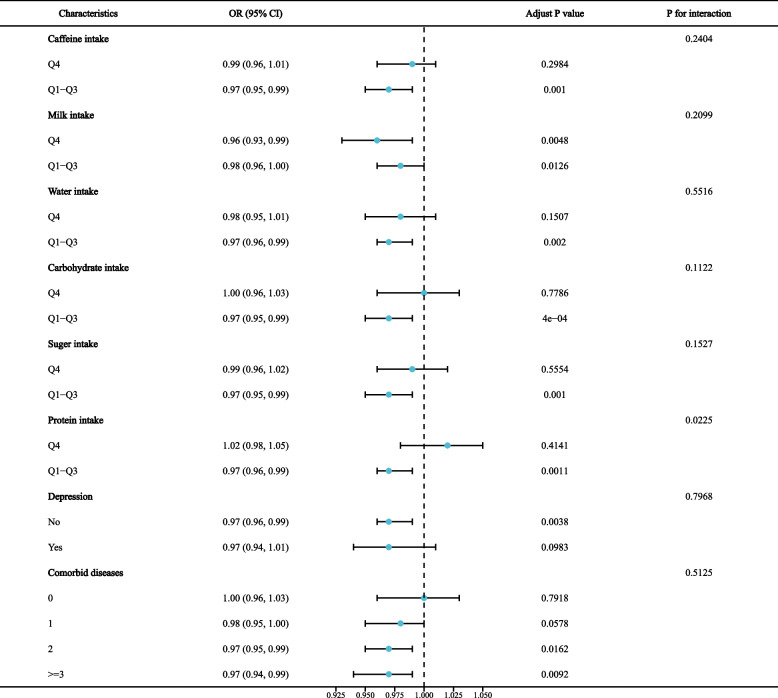
Subgroup analysis for the association between OBS and diarrhea

**Fig. 2:**
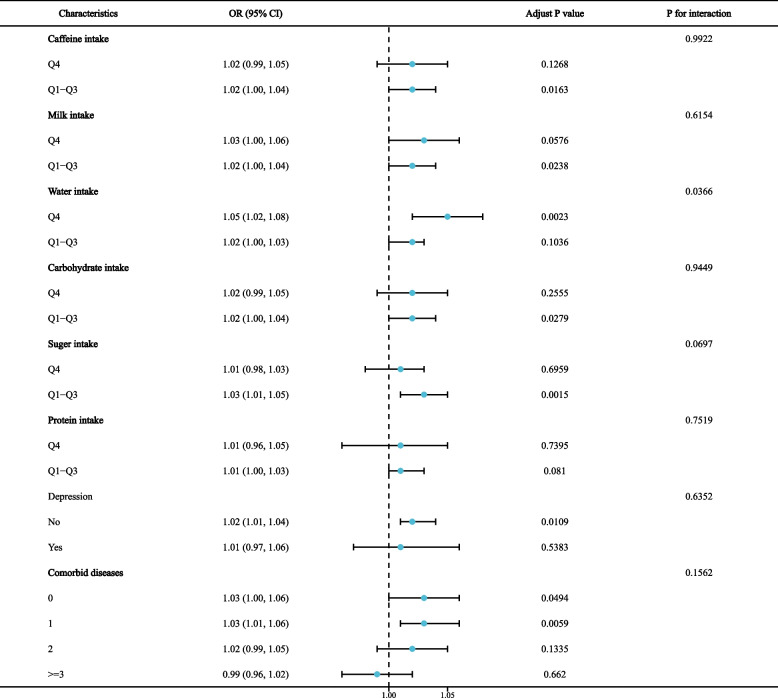
Subgroup analysis for the association between OBS and constipation

Table 3 show the results of the multiple linear regression between OBS and chronic diarrhea and chronic constipation. When OBS was treated as a continuous variable, OBS was negatively associated with diarrhea (OR = 0.98, 95% CI = 0.96–0.99), and these negative associations remained significant after fully adjusting for variables (OR = 0.97, 95% CI = 0.95–0.99). OBS was not significantly associated with constipation according to the original model or Model 1 but was positively associated after fully adjusting for variables (OR = 1.03, 95% CI = 1.01–1.05). When OBS was used as a categorical variable, the highest quartile of OBS was negatively associated with diarrhea compared to the reference group (OR = 0.57, 95% CI = 0.39–0.83). There was no significant association between OBS and constipation in either the original model or Model 1; however, in Model 3, the highest quartile of OBS was positively associated with constipation compared to the reference group (OR: 1.75, 95% CI: 1.19–2.55). All of the above trends were statistically significant (*P* for trend < 0.05). In addition, in Model 2, participants in the highest quartile of dietary OBS versus lifestyle OBS were more likely to have constipation and less likely to have diarrhea (Supplementary Tables 1 and 2). To further validate the experimental results, we performed subgroup analyses. The subgroup analysis of the diarrhea group showed (Fig. 1) that higher OBS was associated with lower odds of diarrhea in patients with low protein intake (*P* = 0.001), but the other subgroup analysis did not reveal a significant effect on the negative association between OBS and diarrhea. Subgroup analysis of constipation group showed (Fig. 2) that higher OBS in patients with low sugar intake was associated with higher constipation rate (*P* = 0.002), but other subgroup analysis did not show significant impact on the negative correlation between OBS and constipation (*P* for interaction > 0.05).

**Table 3:** Association Between OBS with diarrhea and constipation[ Weighted ORs (95%CIs)]

	diarrhea			constipation		
	crude model	Model 1	Model 2	crude model	Model 1	Model 2
OBS	0.98 (0.96, 0.99) *	0.98 (0.97, 1.00)	0.97 (0.95, 0.99) **	0.99 (0.97, 1.01)	1.00 (0.98, 1.02)	1.03 (1.01, 1.05) **
OBS quartile						
Q1	Ref	Ref	Ref	Ref	Ref	Ref
Q2	0.88 (0.65, 1.20)	0.93 (0.69, 1.26)	0.84 (0.63, 1.11)	0.93 (0.70, 1.23)	1.01 (0.75, 1.37)	1.27 (0.93, 1.72)
Q3	0.58 (0.41, 0.83) **	0.64 (0.45, 0.91) *	0.53 (0.38, 0.75) **	0.73 (0.52, 1.03)	0.83 (0.57, 1.19)	1.19 (0.80, 1.78)
Q4	0.66 (0.49, 0.90) *	0.76 (0.56, 1.04)	0.57 (0.39, 0.83) **	0.84 (0.62, 1.14)	1.03 (0.75, 1.42)	1.75 (1.19, 2.55) **
p for trend	0.004	0.038	0.003	0.165	0.930	0.015

Model 1 was adjusted for age, gender, race, education, marital status and PIR

Model 2 was adjusted for age, gender, race, education, marital status and PIR, milk intake, liquid intake, carbohydrates intake, sugar intake, protein intake, caffeine intake, depression, and the number of combined diseases

*OBS* oxidative balance score

^*^P < 0.05

^**^P < 0.01

### Nonlinear association

**Fig. 3:**
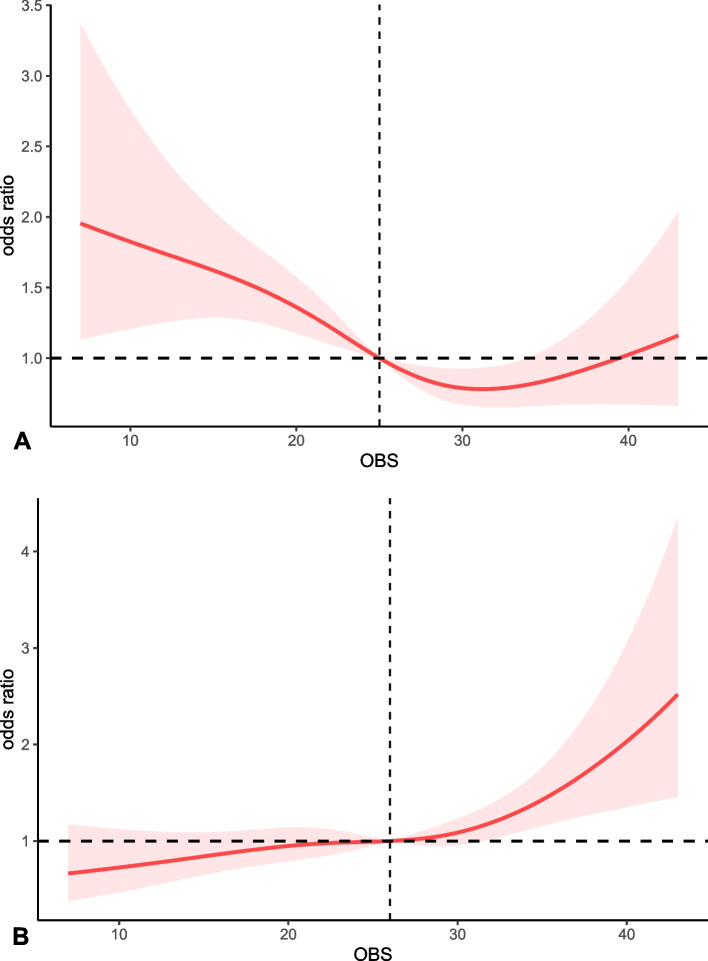
**A** Dose–response associations between OBS and diarrhea. (P for nonlinearity = 0.02). **B** Dose–response associations between OBS and constipation. (P for nonlinearity = 0.19)

To more clearly elucidate the relationship between OBS and diarrhea and constipation, we performed an RCS analysis in the adjustment model (Fig. 3). According to the RCS model, both total OBS and dietary OBS were nonlinearly negatively associated with diarrhea (*P* < 0.05), and lifestyle OBS was linearly negatively associated with diarrhea. Total OBS, dietary OBS, and lifestyle OBS were linearly and positively associated with constipation (*P* < 0.05) ( Supplementary Fig. 2).

### Mediation analyses between OBS and chronic diarrhea and constipation

Mediation analysis showed that the CRP concentration and WBC count significantly mediated the association between OBS and diarrhea, with values of 6.28% and 6.53%, respectively (both *P* < 0.05) (Table 4). However, this study did not identify indicators that significantly mediated the association between OBS and constipation.

**Table 4:** Mediation analyses with separate mediators between OBS and diarrhea

	**Direct effect (average)**	***p*-value**	**Mediation effect (average)**	***p*-value**	**Proportion-mediated (average)**
**Separate mediators**					
GGT	-0.0183(-0.0297,-0.0062)	0.0020	-0.0004(-0.0011,0.0002)	0.1680	-----
CRP	-0.0174(-0.0298,-0.0051)	0.0040	-0.0012(-0.0021,-0.0001)	0.0380	6.276%
WBC	-0.0178(-0.0298,-0.0065)	0.0040	-0.0012(-0.0029,-0.0001)	0.0360	6.532%
Creatinine(mg/dl)	-0.0191(-0.0303,-0.0067)	0.0020	0.0003(-0.0001,0.0010)	0.1260	-----
25(OH)D	-0.0197(-0.0315, -0.0058)	0.0040	-0.0007(-0.0030,0.0013)	0.5320	------

Mediation effect: Effect of mediators on diarrhea

Direct effect: Direct effect of OBS on diarrhea in mediation design

Proportion-mediated: The proportion of people affected by mediating effects

*OBS* oxidative balance score, *GGT* gamma-glutamyl transpeptidase, *CRP* C-reactive protein, *WBC* white blood cells

### Sensitivity analysis

Considering that OBS is composed of multiple components, we explored the relationship between each component and diarrhea and constipation (Supplementary Tables 3 and 4). The results showed that for diarrhea, only BMI was positively correlated with diarrhea in the 20 components; that is, the higher the BMI was, the more likely the subject was to have diarrhea symptoms. The other components were not related to diarrhea. For constipation, according to Model 2, dietary fiber, total fat content and BMI were negatively correlated with constipation; that is, the greater the dietary fiber or total fat content of food or BMI was, the lower the probability of constipation. In addition, our study hypothesized that the relationship between OBS and defecation habits is mediated by oxidative stress. Therefore, we further included the factors mentioned in the literature that may affect the production of ROS in the body via multiple linear regression to exclude the influence of these factors on the results. The factors involved include the use of NSAIDs [33], heavy metals (lead, cadmium) [34] and environmental toxicants (benzene, toluene) [35]. The results of the sensitivity analysis were consistent with those of the main analysis (Table 5). According to the sensitivity analysis, in the fully adjusted logistic regression model, whether OBS was a continuous variable or a categorical variable, high-level OBS was negatively correlated with diarrhea and positively correlated with constipation. The trends were statistically significant (*P* for trend < 0.05).

**Table 5:** Logistic regression analysis on the association between OBS and diarrhea and constipation in the sensitivity analysis

	**diarrhea**			**constipation**		
	**Model 3**	**Model 4**	**Model 5**	**Model 3**	**Model 4**	**Model 5**
OBS	0.97 (0.95, 0.99) **	0.97 (0.95, 0.99) **	0.97 (0.95, 0.99) **	1.03 (1.01, 1.05) **	1.03 (1.01, 1.05) *	1.03 (1.01, 1.05) *
OBS quartile						
Q1	Ref	Ref	Ref	Ref	Ref	Ref
Q2	0.84 (0.63, 1.12)	0.84 (0.63, 1.12)	0.84 (0.63, 1.13)	1.27 (0.93, 1.72)	1.26 (0.93, 1.72)	1.26 (0.93, 1.72)
Q3	0.54 (0.38, 0.76) **	0.54 (0.38, 0.75) **	0.54 (0.38, 0.76) **	1.19 (0.80, 1.78)	1.18 (0.79, 1.77)	1.18 (0.79, 1.78)
Q4	0.58 (0.39, 0.85) **	0.58 (0.39, 0.85) *	0.58 (0.40, 0.85) *	1.74 (1.19, 2.54) **	1.73 (1.16, 2.58) *	1.73 (1.16, 2.58) *
P for trend	0.0036	0.0039	0.0040	0.0155	0.0236	0.0241

Model 3 was adjusted for age, gender, race, education, marital status and PIR, milk intake, liquid intake, carbohydrates intake, sugar intake, protein intake, caffeine intake, depression, the number of combined diseases and NSAIDs

Model 4 was adjusted for age, gender, race, education, marital status and PIR, milk intake, liquid intake, carbohydrates intake, sugar intake, protein intake, caffeine intake, depression, the number of combined diseases, NSAIDs, blood lead and blood cadmium

Model 5 was adjusted for age, gender, race, education, marital status and PIR, milk intake, liquid intake, carbohydrates intake, sugar intake, protein intake, caffeine intake, depression, the number of combined diseases, NSAIDs, blood lead, blood cadmium, blood benzene and blood toluene

OBS: oxidative balance score

^*^P < 0.05

^**^P < 0.01

## Discussion

To our knowledge, this is the first study to assess the relationship between OBS and bowel habits (constipation, diarrhea, and normal) in a nationally representative sample of adults in the U.S. Our study revealed a nonlinear negative correlation between OBS and diarrhea, with CRP and WBC levels mediating this relationship. Interestingly, after adjusting for confounders, OBS was positively associated with constipation, and the RCS plot showed a linear relationship. These findings may provide potential theoretical references for understanding intestinal function from the perspectives of oxidative stress and inflammation as well as preventing oxidative stress-induced changes in intestinal habits. However, previous studies have shown that oxidative stress contributes to the development of constipation by analyzing changes in ROS and superoxide dismutase (SOD) levels in the body [36]. Our study revealed a positive correlation between OBS and constipation, which seems to imply that oxidative stress is a protective factor against constipation, which is inconsistent with the findings of previous studies. Given that OBS is a reflection of overall antioxidant status based on diet and lifestyle, it seems more reasonable to use OBS rather than compounds such as ROS to represent the state of oxidative stress in vivo. However, these results still need to be interpreted with caution.

OBS combines a variety of pro-oxidants and antioxidants in the diet and lifestyle compared to studies focusing on a single nutrient and therefore may be a more accurate indicator of the response to overall oxidative stress. Studies have shown that OBS and its components are associated with reduced risks of cardiovascular disease, colorectal adenoma, and mortality [37]. Previous studies on NHANES data have also confirmed that OBS is closely related to depression, sleep disorders and other diseases [38, 39]. Our study showed that OBS was strongly associated with changes in intestinal habits. It was negatively associated with diarrhea and positively associated with constipation.

There is no direct evidence of an association between OBS and intestinal habits, and how OBS affects changes in intestinal habits is unclear; however, oxidative stress may play an important role in this relationship. Increased oxidative stress was found to play an important role in the age-related decrease in internal anal sphincter tone in aged rats as a potential mechanism for fecal incontinence [40]. An earlier study showed that in the gastrointestinal tract, the colon is more susceptible to damage from oxidative stress, as evidenced by increased apoptosis of colonic neurons and reduced ganglion size [41]. The effects of oxidative stress on colon function (increased nitrogen energic neuromuscular transmission, decreased smooth muscle tone, and altered patterns and parameters of colon motility) mediate constipation in mice treated with oxaliplatin [42]. Few studies have examined the relationship between oxidative stress and diarrhea, but a small number of studies have shown that oxidative stress can lead to diarrhea by triggering intestinal inflammation and injury, causing the release of reactive substances such as NO and oxygen into the intestinal lumen and ultimately leading to the apoptosis of intestinal epithelial cells and ultimately diarrhea [43]. Symptoms of diarrhea and constipation can be improved by improving oxidative stress. Antioxidant compounds, especially polyphenols from plants, can eliminate free radicals and alleviate intestinal disorders associated with oxidative stress [44]. Grape seed procyanidins [45], zinc [46], quercetin [47], and other antioxidants have been found to reduce the incidence of diarrhea by increasing the body's antioxidant capacity. Selenium, an essential trace element in the human body, has a wide range of antioxidant and anti-inflammatory physiological functions and is significantly negatively correlated with chronic constipation [48]. Probiotics and synbiotics can help alleviate gastrointestinal symptoms, improve the emotional state, and regulate bidirectional bowel habits by reducing oxidative stress markers, which means that constipation patients have an increase in bowel movements while diarrhea patients have a decrease in bowel movements [49]. Furthermore, our study found that dietary OBS and lifestyle OBS are closely related to bowel habits. Our study’s findings are consistent with the results of previous studies. A study by Akinori et al. found that the daily intake of broccoli sprouts (rich in sulforaphane, which has antioxidant properties) normalized bowel habits in healthy human subjects [50]. Several studies have investigated the impact of lifestyle factors, such as smoking, on defecation habits, which can cause both constipation and diarrhea [51, 52]. Proper physical exercise can reduce constipation [25].

The link between oxidative stress and bowel habits can be attributed to two main factors: altered intestinal flora and impaired intestinal barrier function. The intestinal flora plays an important role in the development of intestinal diseases. One of the mechanisms of constipation is the dysbiosis of the intestinal flora, which is mainly manifested by a reduction in bacterial diversity and dominant species in the body [53]. Similarly, gut microbes are involved in the pathogenesis of diarrhea. Diarrhea caused by bacterial pathogens such as Shigella, *Clostridium difficile*, and *Escherichia coli* has become a global health problem, especially in developing countries. An imbalance of the intestinal flora can induce oxidative stress. The gut microbiota can influence the level of reactive ROS in the body, thus affecting the state of oxidative stress in the body. Lactobacilli are powerful inducers of reactive oxygen species and can stimulate the cellular production of reactive oxygen species through specific membrane components or secreted factors [54]. Symbiotic and pathogenic bacteria in the intestine tract can also alter cellular ROS levels by regulating mitochondrial activity [55]. Research on sterile animals emphasizes this point. Injecting fecal supernatant from acute ischemic stroke into the colon of sterile mice can increase ROS and MDA levels and downregulate SOD and GSH activity [56]. In contrast, inhibition of the gut microbiome ameliorates age-related oxidative stress in mice [57]. Oxidative stress can also aggravate intestinal flora disorders in the body. When oxidative stress occurs, the intestinal epithelium passively diffuses oxidized products, increasing the oxidative potential and stimulating the growth of aerobic bacteria, which in turn alters the host's cellular composition and metabolic signals [58]. Furthermore, ROS can stimulate the production of IL-6 and other inflammatory factors through the NF-κB pathway [59], which can increase the abundance of parthenogenetic anaerobes and exacerbate intestinal dysbiosis [60]. In addition, antibiotics can alter the abundance and diversity of the intestinal flora by indirectly affecting oxidative stress in the gut [61], which provides evidence that oxidative stress affects the intestinal flora in one way or another. Taken together, these studies suggest that when the body undergoes oxidative stress resulting in intestinal flora disorders, timely regulation of the intestinal flora may be an effective means of ameliorating oxidative stress and further preventing the occurrence of defecation disorders.

Oxidative stress-induced damage to the intestinal barrier may be another potential mechanism through which oxidative stress affects intestinal habits. Intestinal integrity is the fundamental guarantee of intestinal function. Intestinal permeability and mucosal tight junction proteins provide important epithelial barriers and have been shown to play key roles in the pathogenesis of diarrhea and constipation [62, 63]. Occludin, claudins, and ZO-1 are the main intestinal barrier proteins. However, studies have shown that the expression of ZO-1, occludin, and claudin-1 in cells is significantly reduced after H2O2 treatment. Resveratrol can alleviate H2O2-induced cell damage by upregulating the oxidative state, thus alleviating intestinal damage, especially barrier damage [64]. Excess ROS has also been found to alter the integrity of epithelial cells and the intestinal barrier by reducing tight junctions and cell count [65]. Therefore, oxidative stress can cause gastrointestinal symptoms such as diarrhea and constipation by exacerbating intestinal mucosal damage and disrupting the intestinal barrier.

In addition, we explored the potential pathways associated with OBS-related effects on diarrhea and constipation. Unfortunately, our study did not find any mediating effects of OBS on constipation, but mediation analysis revealed that the association between OBS and diarrhea was mediated by the CRP concentration and WBC count, with mediation ratios of 6.28% and 6.53%, respectively. This result strongly supports the hypothesis that the high oxidative stress state reflected by low OBS may exacerbate diarrhea symptoms by inducing inflammation. Consistent with our results, some studies have reported a negative correlation between OBS and inflammatory biomarkers. Sindhu et al [66]. reported a negative correlation between OBS and high CRP and total white blood cell count, while Kong et al. also reported a correlation between higher OBS and lower levels of the inflammatory marker CRP [67]. As a widely recognized biomarker of oxidative stress, GGT levels exhibit a strong negative correlation with OBS. Our study did not find that GGT plays a mediating role in the relationship between OBS and diarrhea, but oxidative stress cannot be considered to play a role in the relationship between OBS and diarrhea, as other biomarkers that respond to oxidative stress, such as F2 isoproteins, were not included in this study due to incomplete data information in the NHANES database. The research results are also affected by many factors, such as the study population, survey methods, and systematic errors. Therefore, additional prospective research is needed to explore the relationship between OBS and changes in bowel habits, as well as the mediating role of oxidative stress.

We analyzed the relationships between OBS components and diarrhea and constipation and found that BMI was positively correlated with diarrhea and negatively correlated with constipation. This finding is consistent with previous research results [68, 69]. Taking Ogasawara N's study as an example, the author studied the relationship between BMI and BSFS score and found that BMI increased from the BSFS-1/2 through the BSFS-3/4/5 and then the BSFS-6/7 groups in both males and females. The relationship between BMI and defecation habits is related to many factors, such as a reduction in rectosigmoid transit time and idiopathic bile acid malabsorption [70, 71]. Our study also revealed that fat and dietary fiber intake were negatively correlated with constipation. This result is not difficult to explain. An increased nutrient content in a high-fat diet leads to intestinal adaptation, such as cell proliferation, increased intestinal length and nutrient uptake. This leads to reduced exposure of the intestinal field to receptors that are activated by fat, such as fat-stimulated ileal mechanisms, leading to slower gastric emptying and small intestinal transit [72]. A high-fat diet may also damage enteric neurons in humans in a similar fashion that is shown in mice via a pathway mediated by microRNA375 upregulation [73]. Dietary fiber has also been found to improve constipation through improved fecal movement content, shortened fecal empty time, reduced serum VIP concentration and other pathways [74]. Increasing dietary fiber intake can significantly reduce medical expenses caused by constipation [75].

This study has several limitations. First, this was a cross-sectional study, and we were unable to determine causal relationships. However, we believe that the discovery of this relationship is highly important for practical clinical and public health practices. For example, healthcare professionals can develop more precise health promotion and disease prevention strategies. In addition, conveying this discovery to patients will help increase their awareness of intestinal health, enabling them to actively participate in self-management and adopt appropriate lifestyle adjustments to improve intestinal health. Second, we used the average of two 24-h reminders to estimate dietary composition. It is not clear whether this short-term indicator can better represent the overall state of oxidative stress within 30 days (data on changes in bowel habits come from the most common bowel state in the last 30 days). Individual dietary patterns may vary in different seasons. However, this research method has a certain degree of scientific validity and has been used in multiple studies. Finally, the indicators of oxidative stress included in this study were insufficient, and the mediating effects obtained were only the result of statistical analysis. Considering the limitations of the current research, caution should be taken when interpreting the results obtained thus far. This study provides a certain direction for future research and therefore more comprehensive clinical trials must be designed to further clarify the relationship between oxidative stress and changes in bowel habits.

## Conclusions

In this study, we found that a higher OBS was negatively correlated with diarrhea and positively correlated with constipation. OBS is an indicator of the body's antioxidant status, so this result may suggest the role of oxidative stress in the change of defecation habits and plays an important role in clinical practice and public health practice. However, prospective and experimental studies are still needed in the future to verify this association and its potential underlying mechanisms.

### Supplementary Information

**Supplementary Material 1.**

## Data Availability

More information about the NHANES could be obtained at: http://www.cdc.gov/nhanes.

## Abbreviations

OBS: Oxidative balance score
PIR: Poverty income ratio
RCS: Restricted cubic spline
24HR: 24-Hour dietary recall interviews
BSFS: Bristol stool form scale
ROS: Reactive oxygen species
CRP: C-reactive protein
WBC: White blood cells
GGT: Gamma-glutamyl transferase
SOD: Superoxide Dismutase
MDA: Malondialdehyde
BMI: Body mass index
CDC: Centers for Disease Control and Prevention
