# Supplementary material for: The relationship between oxidative balance scores and chronic diarrhea and constipation: a population-based study
Source: BMC Public Health. 2024 May 21;24:1366. doi: 10.1186/s12889-024-18683-8 (PMC11106991; doi:10.1186/s12889-024-18683-8)
Supplement: Supplementary file 1 — Supplementary Material 1. [file 12889_2024_18683_MOESM1_ESM.pdf]

The relationship between Oxidative balance scores and chronic diarrhea and constipation: A population-based study

Jiayan Hu [1,2] , Hede Zou[1], Xiyun Qiao [1,2] , Yuxi Wang [1,2,3], Mi Lv [1,2] , Kunli Zhang [1,2], Fengyun Wang [1,2*]

(1. Xiyuan Hospital of China Academy of Chinese Medical Sciences, Beijing, China

2.Institute of Digestive Diseases, Xiyuan Hospital of China Academy of Chinese Medical Sciences, Beijing, China

3. Beijing University of Chinese Medicine.)

[*]Correspondence

Fengyun Wang, M.D, Ph.D. Professor, Xiyuan Hospital of Chinese Academy of Chinese Medical Sciences. Beijing 100091, People's Republic of China   E-Mail:wfy811@163.com

Supplementary Table 1. Association Between OBS dietary/ OBS lifestyle with diarrhea [Weighted ORs (95%CIs)]

	crude model	Model 1	Model 2
OBS. dietary	0.98 (0.96, 1.00) *	0.99 (0.97, 1.01)	0.97 (0.95, 0.99) *
OBS. dietary quartile			
Q1	Ref.	Ref.	Ref.
Q2	0.85 (0.62, 1.15)	0.90 (0.66, 1.22)	0.78 (0.57, 1.07)
Q3	0.65 (0.45, 0.93) *	0.72 (0.50, 1.04)	0.57 (0.39, 0.83) **
Q4	0.68 (0.49, 0.95) *	0.78 (0.56, 1.10)	0.54 (0.36, 0.81) **
p for trend	0.020	0.116	0.004
OBS. lifestyle	0.88 (0.83, 0.94) **	0.90 (0.84, 0.95) **	0.92 (0.86, 0.98) *
OBS. lifestyle quartile			
Q1	Ref.	Ref.	Ref.
Q2	1.00 (0.74, 1.36)	1.05 (0.77, 1.42)	1.08 (0.80, 1.46)
Q3	0.67 (0.47, 0.97) *	0.70 (0.48, 1.02)	0.73 (0.50, 1.07)
Q4	0.58 (0.43, 0.78) **	0.62 (0.46, 0.83) **	0.67 (0.50, 0.92) *
p for trend	＜0.001	＜0.001	0.005

*p<0.05; **p<0.01

Model 1 was adjusted for age, gender, race, education, marital status and PIR.

Model 2 was adjusted for age, gender, race, education, marital status and PIR, milk intake, liquid intake, carbohydrates intake, sugar intake, protein intake, caffeine intake, depression, and the number of combined diseases.

OBS: oxidative balance score

Supplementary Table 2. Association Between OBS dietary/ OBS lifestyle with constipation [Weighted ORs (95%CIs)]

	crude model	Model 1	Model 2
OBS. dietary	0.99 (0.97, 1.00)	0.99 (0.98, 1.01)	1.03 (1.01, 1.05) *
OBS. dietary quartile			
Q1	Ref.	Ref.	Ref.
Q2	0.86 (0.63, 1.16)	0.92 (0.68, 1.26)	1.17 (0.83, 1.64)
Q3	0.76 (0.56, 1.04)	0.84 (0.61, 1.16)	1.24 (0.86, 1.80)
Q4	0.79 (0.57, 1.09)	0.93 (0.68, 1.29)	1.69 (1.12, 2.55) *
p for trend	0.105	0.566	0.017
OBS. lifestyle	1.03 (0.96, 1.10)	1.09 (1.02, 1.18) 0.0206	1.11 (1.03, 1.19) *
OBS. lifestyle quartile			
Q1	Ref.	Ref.	Ref.
Q2	1.06 (0.76, 1.48)	1.17 (0.84, 1.63)	1.16 (0.83, 1.64)
Q3	1.03 (0.79, 1.34)	1.19 (0.91, 1.56)	1.20 (0.91, 1.57)
Q4	1.15 (0.88, 1.51)	1.49 (1.12, 1.97) **	1.54 (1.16, 2.05) **
p for trend	0.371	0.020	0.011

*$p<0.05$; **$p<0.01$

Model 1 was adjusted for age, gender, race, education, marital status and PIR.

Model 2 was adjusted for age, gender, race, education, marital status and PIR, milk intake, liquid intake, carbohydrates intake, sugar intake, protein intake, caffeine intake, depression, and the number of combined diseases.

OBS: oxidative balance score

Supplementary Table 3. The relationship between OBS components and diarrhea.

	**Crude Model**	**Model 1**	**Model 2**
Dietary fiber (g/d)	0.99 (0.97, 1.01)	1.00 (0.98, 1.02)	1.00 (0.97, 1.03)
Total fat (g/d)	1.00 (0.99, 1.00)	1.00 (1.00, 1.01)	1.00 (1.00, 1.01)
Riboflavin (mg/d)	0.97 (0.86, 1.10)	1.07 (0.95, 1.21)	1.03 (0.87, 1.23)
Niacin (mg/d)	0.99 (0.98, 1.00)	1.01 (0.99, 1.02)	1.00 (0.99, 1.01)
Vitamin B6 (mg/d)	0.94 (0.85, 1.05)	1.03 (0.93, 1.14)	1.02 (0.93, 1.12)
Total folate (mcg/d)	1.00 (1.00, 1.00)	1.00 (1.00, 1.00)	1.00 (1.00, 1.00)
Vitamin B12 (mcg/d)	1.00 (0.97, 1.02)	1.01 (0.99, 1.02)	1.00 (0.98, 1.02)
Vitamin C (mg/d)	1.00 (1.00, 1.00)	1.00 (1.00, 1.00)	1.00 (1.00, 1.00)
Vitamin E (ATE) (mg/d)	0.99 (0.96, 1.02)	1.01 (0.98, 1.04)	1.00 (0.98, 1.04)
Calcium (mg/d)	1.00 (1.00, 1.00)	1.00 (1.00, 1.00)	1.00 (1.00, 1.00)
Magnesium (mg/d)	1.00 (1.00, 1.00)	1.00 (1.00, 1.00)	1.00 (1.00, 1.00)
Iron (mg/d)	0.99 (0.97, 1.01)	1.00 (0.98, 1.02)	1.00(0.98, 1.02)
Zinc (mg/d)	0.99(0.97, 1.00)	1.00 (0.99, 1.01)	0.99 (0.97, 1.00)
Copper (mg/d)	1.01 (0.87, 1.18)	1.09 (0.98, 1.20)	1.07 (0.97, 1.18)
Selenium (mcg/d)	1.00 (1.00, 1.00)	1.00 (1.00, 1.00)	1.00 (0.99, 1.01)
Alcohol (drinks/d)	1.00 (0.99, 1.00)	1.00 (1.00, 1.01)	1.00 (1.00, 1.01)
Body mass index (kg/m2)	1.04 (1.03, 1.05) **	1.04 (1.02, 1.05) **	1.03 (1.02, 1.05) **
Physical activity (MET-minute/week)	1.00 (1.00, 1.00)	1.00 (1.00, 1.00)	1. 00 (1.00, 1.00)
Cotinine (ng/mL)	1.00 (1.00, 1.00)	1.00 (1.00, 1.00)	1.00 (1.00, 1.00)
Carotene (RE/d)	1.00 (1.00, 1.00)	1.00 (1.00, 1.00)	1.00 (1.00, 1.00)

*P<0.05; **P<0.01

Model 1 was adjusted for age, gender, race, education, marital status and PIR.

Model 2 was adjusted for age, gender, race, education, marital status and PIR, milk intake, liquid intake, carbohydrates intake, sugar intake, protein intake, caffeine intake, depression, and the number of combined diseases.

OBS: oxidative balance score

Supplementary Table 4.The association between OBS components and constipation.

	**Crude Model**	**Model 1**	**Model 2**
Dietary fiber (g/d)	0.96 (0.94, 0.97) **	0.97 (0.96, 0.99) **	0.9761 (0.9575, 0.9950) *
Total fat (g/d)	0.99 (0.99, 0.99) **	0.9946 (0.9918, 0.9974) **	0.9927 (0.9882, 0.9973) **
Riboflavin (mg/d)	0.75 (0.66, 0.86) **	0.92 (0.82, 1.03)	0.98 (0.83, 1.16)
Niacin (mg/d)	0.97 (0.96, 0.98) **	1.00 (1.00, 1.00)	1.00 (0.98, 1.01)
Vitamin B6 (mg/d)	0.75 (0.68, 0.83) **	0.90 (0.83, 0.99) *	0.98 (0.90, 1.07)
Total folate (mcg/d)	0.9986 (0.9980, 0.9992) **	1.00 (1.00, 1.00)	1.00 (1.00, 1.00)
Vitamin B12 (mcg/d)	0.96 (0.92, 0.99) *	0.99 (0.97, 1.03)	1.01 (0.99, 1.02)
Vitamin C (mg/d)	1.00 (1.00, 1.00)	1.00 (1.00, 1.00)	1.00 (1.00, 1.00)
Vitamin E (ATE) (mg/d)	0.94 (0.92, 0.97) **	0.9741 (0.9523, 0.9965) *	0.99 (0.96, 1.01)
Calcium (mg/d)	0.9995 (0.9993, 0.9998) **	1.00 (1.00, 1.00)	1.00 (1.00, 1.00)
Magnesium (mg/d)	0.9971 (0.9961, 0.9981) **	1.00 (1.00, 1.00)	1.00 (1.00, 1.00)
Iron (mg/d)	0.96 (0.94, 0.97) **	0.98 (0.97, 1.00)	0.99 (0.96, 1.01)
Zinc (mg/d)	0.94 (0.92, 0.96) **	0.97 (0.95, 0.99) **	0.97 (0.94, 1.01)
Copper (mg/d)	0.63 (0.49, 0.81) **	0.86 (0.70, 1.06)	1.01 (0.88, 1.17)
Selenium (mcg/d)	0.9928 (0.9893, 0.9964) **	1.00 (0.99, 1.00)	1.00 (0.99, 1.01)
Alcohol (drinks/d)	0.99 (0.98, 0.99) **	0.9913 (0.9830, 0.9997) *	0.99 (0.98, 1.00)
Body mass index (kg/m2)	0.9752 (0.9549, 0.9960) *	0.98(0.96, 0.99) *	0.98 (0.96, 0.99) *
Physical activity (MET-minute/week)	1.00 (1.00, 1.00)	1.00 (1.00, 1.00)	1.00 (1.00, 1.00)
Cotinine (ng/mL)	1.00 (1.00, 1.00)	1.00 (1.00, 1.00)	1.00 (1.00, 1.00)
Carotene (RE/d)	1.00 (1.00, 1.00)	1.00 (1.00, 1.00)	1.00 (1.00, 1.00)

*P<0.05; **P<0.01

Model 1 was adjusted for age, gender, race, education, marital status and PIR.

Model 2 was adjusted for age, gender, race, education, marital status and PIR, milk intake, liquid intake, carbohydrates intake, sugar intake, protein intake, caffeine intake, depression, and the number of combined diseases.

OBS: oxidative balance score

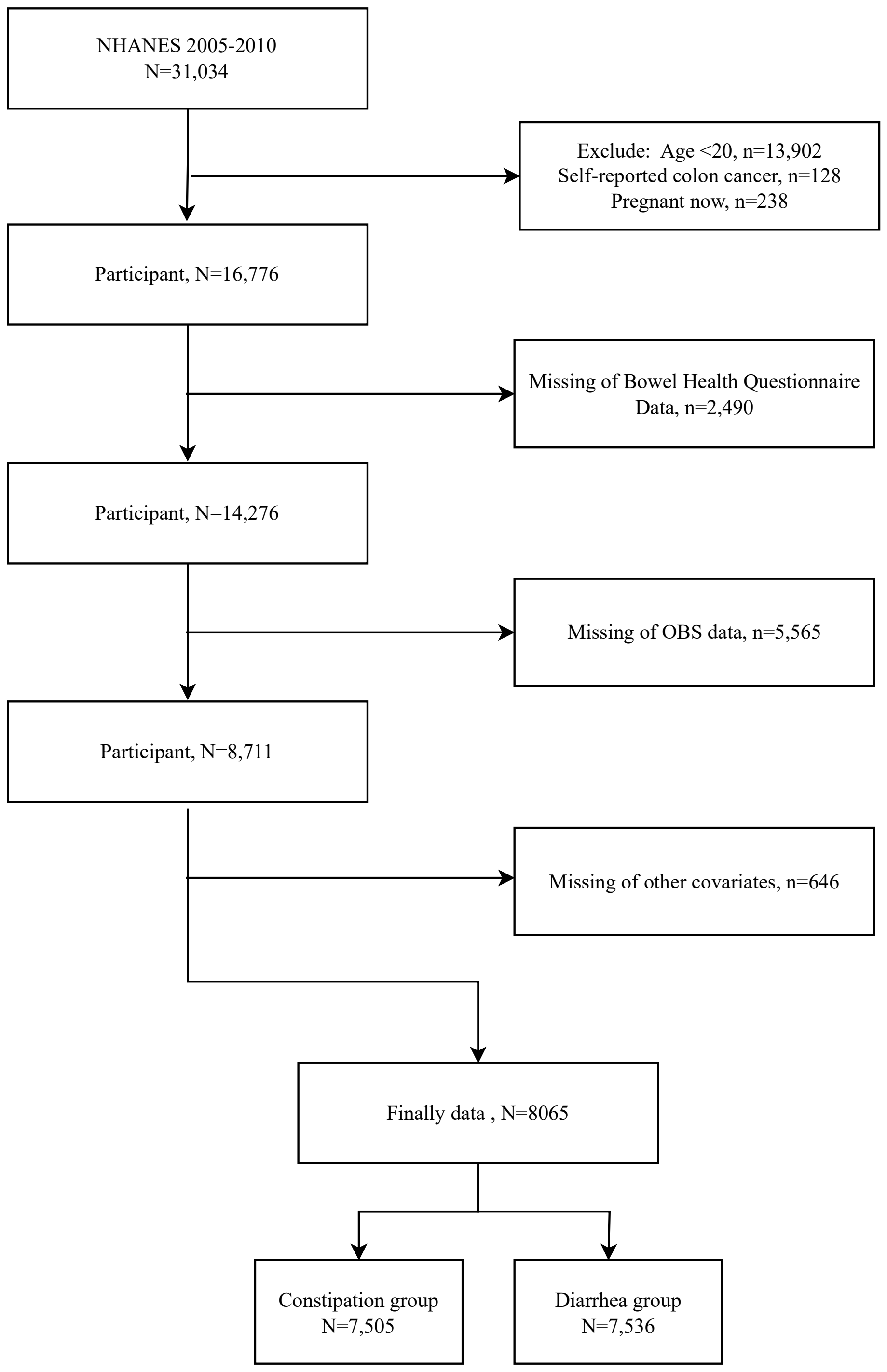

Supplementary Figure 1. Flowchart of the sample selection from NHANES 2005–2010

A

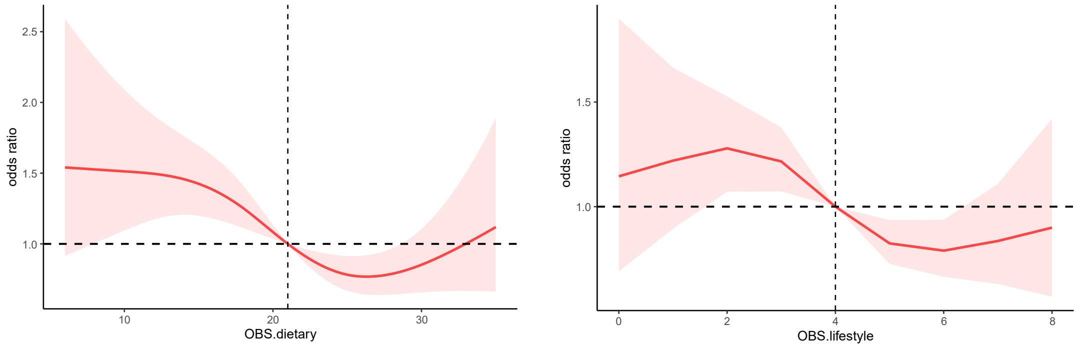

B

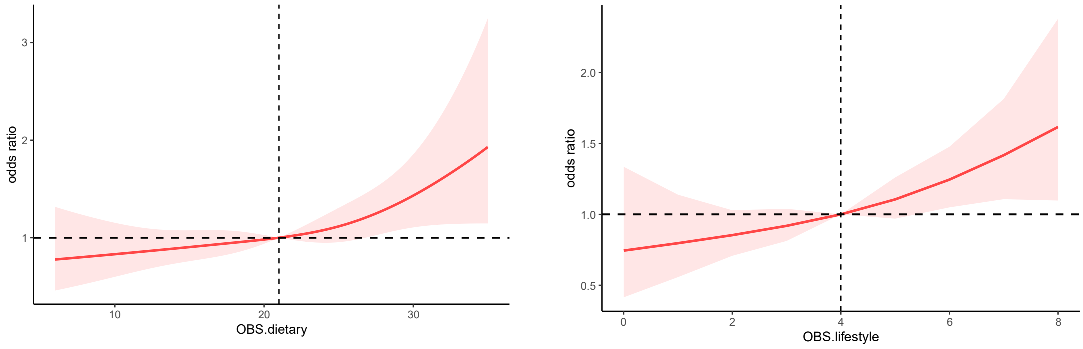

Supplementary Figure 2. (a).Dose-response associations between OBS dietary/OBS lifestyle and diarrhea. (*p* for nonlinearity = 0.03、*p* for nonlinearity = 0.16 respectively). (b) Dose-response associations between OBS dietary/OBS lifestyle and constipation. (*p* for nonlinearity = 0.45、*p* for nonlinearity = 0.87 respectively).

Supplemental Methods. The combined disease criteria

**Diseases**	**Diagnostic Criteria**
Depression	Patient Health Questionnaire (PHQ-9)≥10.
Stroke	When subjects answered "yes" to the question "Has a doctor or other health professional ever told you that you had a stroke?", they were considered to have a stroke.
Coronary Heart Disease	When subjects answered "yes" to the question "whether a doctor or other health professional has ever told you that you had CHD", they were considered to have CHD.
Chronic Kidney Disease	eGFR < 60 mL/min/1.73 m2 and ACR <30 mg/g
Diabetes	(1) Physician diagnosis of diabetes. (2) Glycated hemoglobin HbA1c (%) >6.5. (3) Fasting blood sugar (mmol/L) >7.0.4. (4) Random blood sugar (mmol/L) ≥11.1. (5) 2-hour OGTT blood sugar (mmol/L) ≥11.1. (6)Use of diabetes medications or insulin.
Hypertension	(1)When subjects answered "yes" to the question "Have you ever been told by a doctor or other health professional that you have hypertension?".(2) Self-reported antihypertensive drug use.(3) A high biological measurement value (systolic blood pressure ≥140 mm Hg and/or diastolic blood pressure ≥90 mm Hg).<br>The average blood pressure was calculated by the following protocol: The diastolic reading with zero is not used to calculate the diastolic average. If all diastolic readings were zero, then the average would be zero. If only one blood pressure reading was obtained, that reading is the average. If there is more than one blood pressure reading, the first reading is always excluded from the average.
Hyperlipidemia	(1)total cholesterol ≥200 mg/dL, HDL 40 mg/dL in males and 50 mg/dL in females, or low-density lipoprotein 130 mg/dL. (2) triglycerides 150 mg/dL . (3)Self-reported using cholesterol-lowering drugs.
